# Predictors of Early (0–7 Days) and Late (8–30 Days) Readmission in a Cohort of Acute Coronary Syndrome Patients

**DOI:** 10.5195/ijms.2022.1058

**Published:** 2022

**Authors:** George Cholack, Joshua Garfein, Rachel Krallman, Delaney Feldeisen, Daniel Montgomery, Eva Kline-Rogers, Geoffrey D. Barnes, Kim Eagle, Melvyn Rubenfire, Sherry Bumpus

**Affiliations:** 1 Medical student, MSc. Michigan Medicine, Department of Internal Medicine, Division of Cardiovascular Medicine, Ann Arbor, MI; Oakland University William Beaumont School of Medicine, Rochester, MI, United States.; 2 MPH. Michigan Medicine, Department of Internal Medicine, Division of Cardiovascular Medicine, Ann Arbor, MI, United States.; 3 BS. Michigan Medicine, Department of Internal Medicine, Division of Cardiovascular Medicine, Ann Arbor, MI, United States.; 4 BA. Michigan Medicine, Department of Internal Medicine, Division of Cardiovascular Medicine, Ann Arbor, MI, United States.; 5 NP. Michigan Medicine, Department of Internal Medicine, Division of Cardiovascular Medicine, Ann Arbor, MI, United States.; 6 MD, MSc. Michigan Medicine, Department of Internal Medicine, Division of Cardiovascular Medicine, Ann Arbor, MI, United States.; 7 MD. Michigan Medicine, Department of Internal Medicine, Division of Cardiovascular Medicine, Ann Arbor, MI, United States.; 8 PhD, FNP-BC. Michigan Medicine, Department of Internal Medicine, Division of Cardiovascular Medicine, Ann Arbor, MI; Eastern Michigan University, College of Health and Human Services, School of Nursing, Ypsilanti, MI, United States.

**Keywords:** Myocardial infarction, Unstable angina, Atrial fibrillation, Intensive care unit, Heart failure, Patient readmission (Source: MeSH-NLM)

## Abstract

**Background:**

Readmissions following acute coronary syndrome are unevenly distributed across the 30-day post-discharge period. There is limited data on predictors of all-cause readmission in early (0–7 day) and late (8–30 day) post-discharge periods for this population; the purpose of this retrospective cohort study was to identify predictors of early and late readmission.

**Methods:**

Patients at Michigan Medicine (Ann Arbor, Michigan, United States) with a principal discharge diagnosis of unstable angina, ST-segment elevation myocardial infarction, or non-ST segment elevation myocardial infarction between April 2008 and November 2017 were identified. Predictors of early and late readmission were analyzed with multivariable logistic regression models.

**Results:**

Of 1120 patients hospitalized following acute coronary syndrome, 198 (17.68%) were readmitted within 30 days while 70 (6.25%) were readmitted within 7 days of discharge. Of 30-day readmissions, early readmissions were more likely in females [OR 2.26, 95% confidence interval (CI) 1.23, 4.16], non-white individuals (p=0.05), or patients requiring intensive care unit admission during hospitalization (OR 2.20, 95% CI 1.14, 4.24). Relative to patients not readmitted within 7 days, patients who were female, had history of atrial fibrillation, principal discharge diagnosis of non-ST segment elevation myocardial infarction, or required intensive care unit admission were more likely readmitted early. History of congestive heart failure was a predictor of late readmission when compared to patients not readmitted in 30 days.

**Conclusion:**

Following acute coronary syndrome, predictors of readmission varied between early and late readmission groups. Readmission predictors provides healthcare providers with information useful in minimizing readmissions and concomitant financial penalties.

## Introduction

Readmissions following an acute coronary syndrome (ACS) are not evenly distributed across the 30-day post-discharge period. To date, there is a dearth of research investigating predictors of early (0–7 day) and late (8–30 day) readmission in patients hospitalized for various types of acute coronary syndromes [unstable angina, ST-segment elevation myocardial infarction (STEMI), or non-ST segment elevation myocardial infarction (NSTEMI)]. Studies have shown that among patients with acute myocardial infarction, the majority of these 30-day readmissions occur within the first 14 days post-discharge, and a significant proportion occur within the first 7 days post-discharge.^1–4^ Furthermore, Graham et al.^5,6^ suggest that readmissions within one week of discharge may be amenable to prevention. Other research investigating early (0–7 days) versus late (8–30 days) readmissions in general medicine and heart failure patients, suggests that unique subsets of characteristics may predict whether patients are more likely to be readmitted in the early or late readmission period.^6,7^ Understanding why patients are readmitted at varying points during the 30-day post-discharge period is crucial for minimizing 30-day readmission rates, especially since hospitals with risk-adjusted readmission rates greater than average readmission rates following most ACS hospitalizations incur financial penalties through the Center for Medicare and Medicaid Services’ Hospital Readmission Reduction Program.^8^ While Dharmarajan et al.^1^ illustrated that timing of 30-day readmissions following hospitalization for acute myocardial infarction did not vary substantively by age, sex, or race, it has yet to be investigated if there are predictors associated with differential risk of all-cause readmission in the early and late post-discharge periods following hospitalization for unstable angina, STEMI, or NSTEMI. As early readmissions may be more preventable, such predictors could be helpful to health systems aiming to maximally reduce 30-day readmission rates following ACS hospitalizations.

At Michigan Medicine (Ann Arbor, Michigan, United States), Bridging the Discharge Gap Effectively (BRIDGE), is a transitional care cardiology program with the purpose of reducing hospital readmission rates by ensuring that patients admitted with a cardiac diagnosis are seen by a nurse practitioner within 14 days post-discharge.^9^ Despite overall lower 30-day readmission rates among BRIDGE attendees relative to non-attenders, roughly 50% of readmissions for patients with an index hospitalization of ACS occurred within 14 days of discharge.^9^ Since ACS patients readmitted early often required rehospitalization prior to being able to attend BRIDGE, the purpose of this study was to identify clinical predictors of early and late all-cause readmission in this population.

## Methods

### Bridge Registry

The BRIDGE registry is a retrospective dataset of cardiac patients discharged from Michigan Medicine and referred to the BRIDGE clinic due to lack a scheduled cardiac follow-up within 14 days of discharge.^10^ Details of the registry have been described elsewhere.^9^ Briefly, the BRIDGE registry is a de-identified clinical database consisting of consecutive data that are extracted from an electronic medical record for all patients referred to the BRIDGE clinic. Data are abstracted manually by trained data abstractors, and 10% of all abstractions are audited for accuracy. Abstracted data includes patient demographics, past medical history, index admission and discharge data, and follow-up data within 6 months of index admission. This study was carried out in accordance with the principles outlined in the Declaration of Helsinki and its later amendments. The Human Subjects Internal Review Board of Michigan Medicine approved this study (HUM00035421) with a waiver of informed consent.

### Study Population and Outcomes

**Figure 1.**
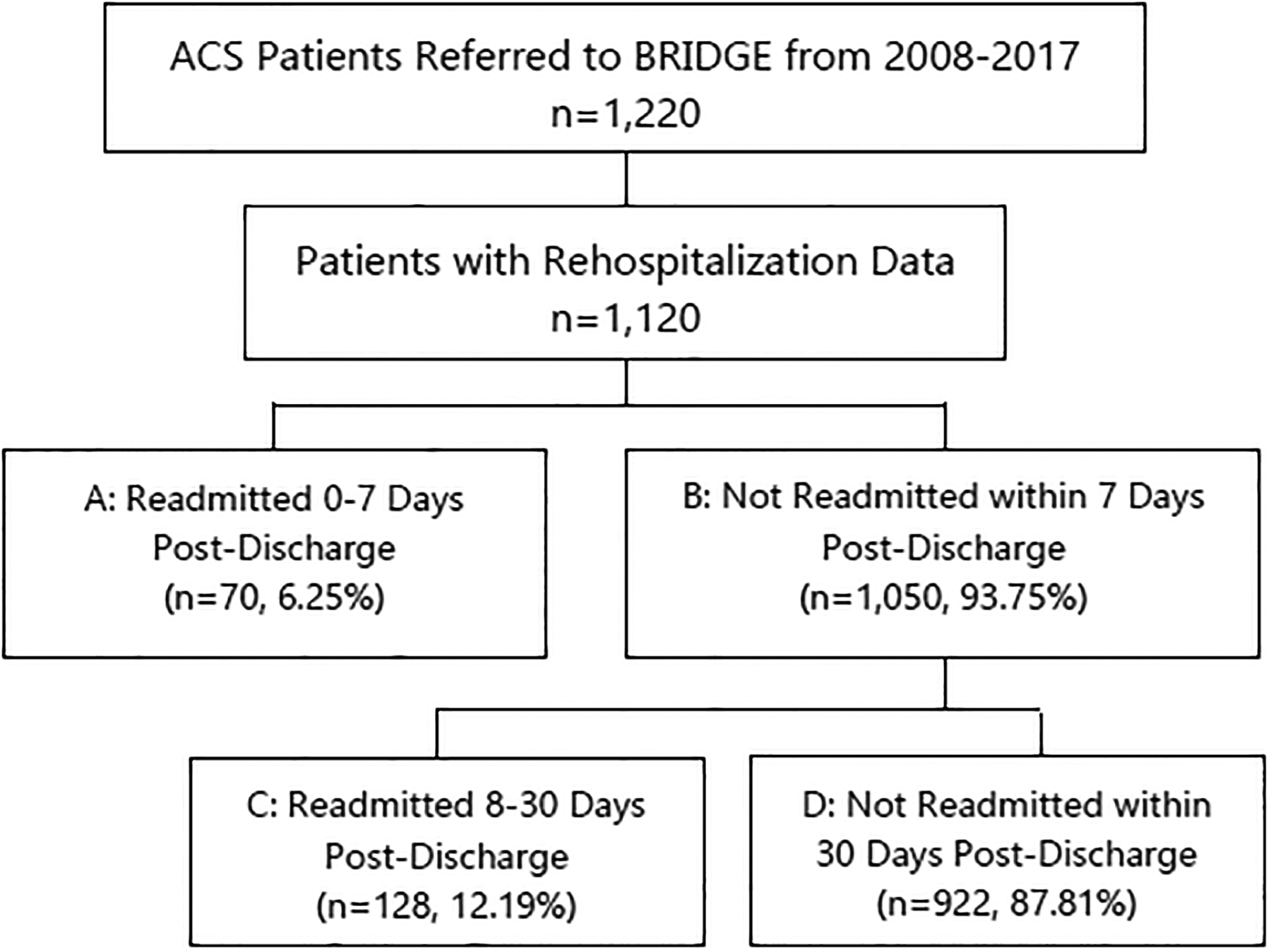
Patient Flow Diagram. ACS (Acute coronary syndrome) Patients Referred to Bridging the Discharge Gap Effectively (BRIDGE) transitional care cardiology program were dichotomized into readmissions within 0–7 days post-discharge, and those not readmitted within 7 days. The latter group was further dichotomized into readmissions 8–30 days post-discharge and those not readmitted within 30 days post-discharge. Percentages in boxes A and B are fractions of the total number of ACS patients referred to BRIDGE who had rehospitalization data. Percentages in boxes C and D are percentages of ACS patients not readmitted within 7 days.

For this retrospective cohort study, patients enrolled in the BRIDGE registry between April 2008 and November 2017 were identified if they had a principal discharge diagnosis of ACS, which included: unstable angina, STEMI, and NSTEMI. The primary outcome of this study was time to readmission for any cause. Patients with unknown readmission status secondary to loss to follow-up were excluded. A separate analysis was conducted to identify the reasons for readmission, which were categorized as recurrent ACS, congestive heart failure (CHF), “other cardiac diagnosis”, or “other non-cardiac diagnosis.” Patients were stratified by time to readmission within 30 days and compared by demographics, specific ACS principal discharge diagnosis (unstable angina, STEMI, NSTEMI), past medical history, readmission diagnosis, admission index factors (ICU admit during index, length of index stay, lab values during index admission), and mortality at 180 days post-discharge (Figure 1):

1. Early Readmissions (0–7 days) vs. Late Readmissions (8–30 days) (Boxes A vs. C)
2. Early Readmissions (0–7 days) vs. No Readmission within 7 days (Boxes A vs. B)
3. Late Readmissions (8–30 days) vs. No Readmission within 30 days (Boxes C vs. D)

### Statistical Analysis

Data were analyzed using SPSS (IBM SPSS Statistics for Windows, Version 25.0. Armonk, New York: IBM Corporation). To compare early and late readmission groups, chi-square and two-tailed Mann-Whitney tests were used for categorical and continuous variables, respectively. Cases with missing data were excluded from analyses. Number of patients in each readmission subgroup with missing data for given variables are provided in Supplemental Tables 2 and 3 Since individual-level data were not available for income or education, the median household income^11^ of each patient’s zip code (based on a five-year average ending in 2016) was used as a proxy for socioeconomic status. Patients were considered “low socioeconomic status” if their residence was located in a zip code associated with a median household income less than the median household income for the State of Michigan. For the Charlson Comorbidity Index (CCI), a modified score was calculated based on similar assumptions utilized by Chang et al.^12^ Admission to the intensive care unit (ICU) was based on the clinical needs of the patient as assessed both by the ICU physicians and nursing staff. Typically, this includes patients who required advanced respiratory or hemodynamic support as well as patients within the first 24 hours after a STEMI. A priori significance was set to α=0.05. Multivariable logistic regression models were then created to identify potential independent predictors of early and late readmission. Bivariate analysis was first utilized to help identify variables as candidates for the models. After considering clinical relevance, variables with bivariate p-values ≤ 0.15 were considered for introduction to the model. A backward stepwise regression method was utilized as an aid to develop the final models. A Hosmer-Lemeshow test was used to determine goodness of fit, and the area under the ROC curve (C-statistic) was computed to describe the discriminatory power of each model. Odds ratios reported in final models are adjusted for all predictors retained in the model.

## Results

### Early Readmissions vs. Late Readmissions

**Figure 2.**
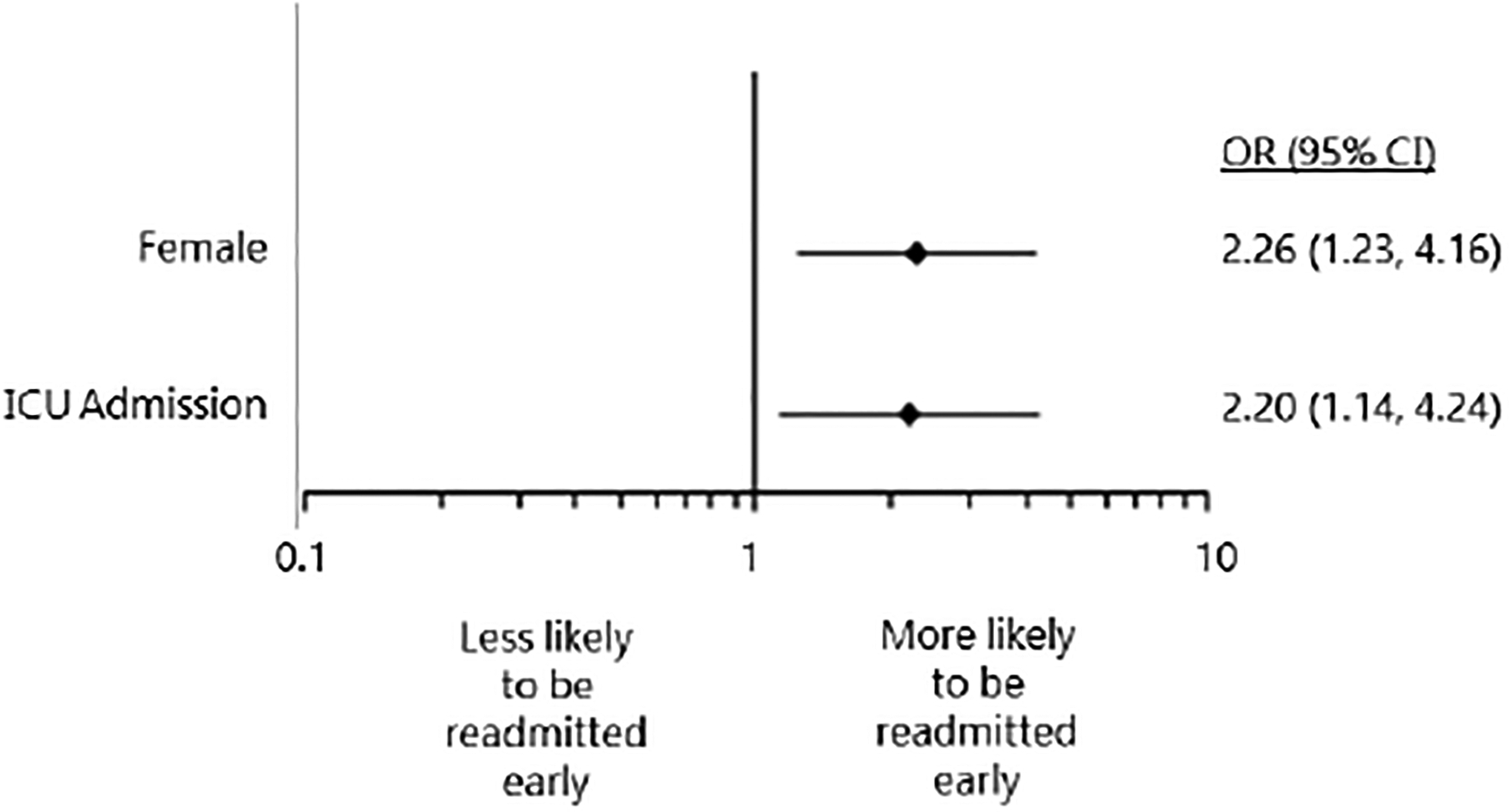
Multivariable Logistic Regression Model Comparing Early (0–7 Days) vs Late (8–30 Days) Readmissions. Late readmissions was used as the reference group. Reported odds ratios are adjusted for sex and ICU admission. Abbreviations: ICU = intensive care unit; OR = odds ratio; CI = confidence interval.

Of 4879 patients referred to BRIDGE from April 2008 to November 2017, 1220 (25.4%) had a principal discharge diagnosis of ACS. Of 1120 ACS patients with rehospitalization data, 198 (17.7%) had an unplanned readmission within 30 days post-discharge (Figure 1). Of 30-day readmissions, 70 (35.4%) were readmitted early (Supplemental Table 1). Relative to late (8–30 day) readmissions, early readmissions (0–7 days) were more likely to be in females, non-white individuals, or patients requiring ICU admission during index hospitalization (Supplemental Table 1). No other differences between early and late readmission groups were observed with respect to demographics, type of ACS (unstable angina, NSTEMI, STEMI), past medical history, readmission diagnosis, other patient characteristics during index hospitalization, and all-cause mortality at 180 days post-discharge (Supplemental Table 1). Readmission diagnoses for early and late readmissions are provided in Table 1 and Supplemental Table 1. Briefly, “other non-cardiac diagnosis” and “other cardiac diagnosis” were the predominant readmission diagnoses for early and late readmissions, respectively. After adjustment, female sex [OR 2.26, 95% confidence interval (CI) 1.23, 4.16], and index intensive care unit (ICU) admission (OR 2.20, 95% CI 1.14, 4.24) were all significant independent predictors of early readmission (C-statistic=0.633 and Hosmer-Lemeshow p-value = 0.78, Figure 2).

#### Early Readmissions vs. No Readmissions in 7 days

**Figure 3.**
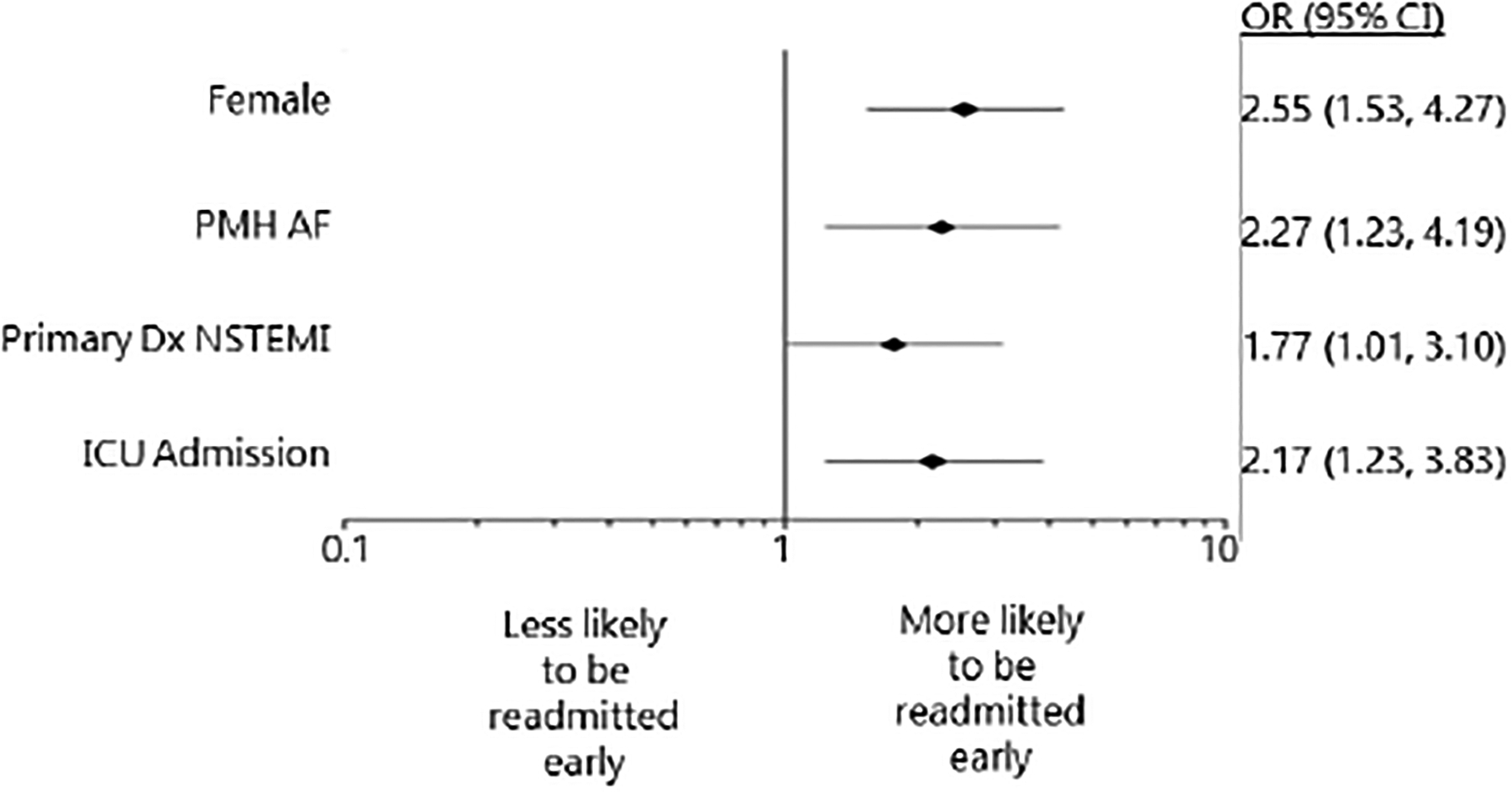
Multivariable Logistic Regression Model Comparing Early Readmission versus No Readmission in Seven Days (Reference Group). Reported odds ratios are adjusted for sex, history of AF, principal discharge diagnosis of NSTEMI, and ICU admission. Abbreviations: PMH = past medical history; AF = atrial fibrillation; Dx = diagnosis; NSTEMI = non-ST segment elevation myocardial infarction; ICU = intensive care unit; OR = odds ratio; CI = confidence interval.

Of 1120 ACS patients with rehospitalization data, 70 (6.25%) were readmitted early (Table 1). Compared to patients not readmitted within the first 7 days, patients readmitted early were significantly older (roughly 4 years on average), more likely female, more likely to have history of atrial fibrillation, or have greater CCI scores. Furthermore, patients readmitted early were more likely to have been in the ICU during their index admission, have a longer overall hospital length of stay, present with lower hemoglobin and higher blood urea nitrogen levels upon index admission, and experience higher all-cause mortality over 180 days post-discharge (Table 1). After adjustment, female sex (OR 2.55, 95% CI 1.53, 4.27), past medical history of atrial fibrillation (OR 2.27, 95% CI 1.23, 4.19), principal discharge diagnosis of NSTEMI (OR 1.77, 95% CI 1.01, 3.10), and index ICU admission (OR 2.17, 95% CI 1.23, 3.83) were all significant independent predictors of early readmission among all ACS patients studied (C-statistic=0.677 and Hosmer-Lemeshow p-value = 0.91, Figure 3).

### Late Readmissions vs. No Readmissions in 30 days

**Figure 4.**
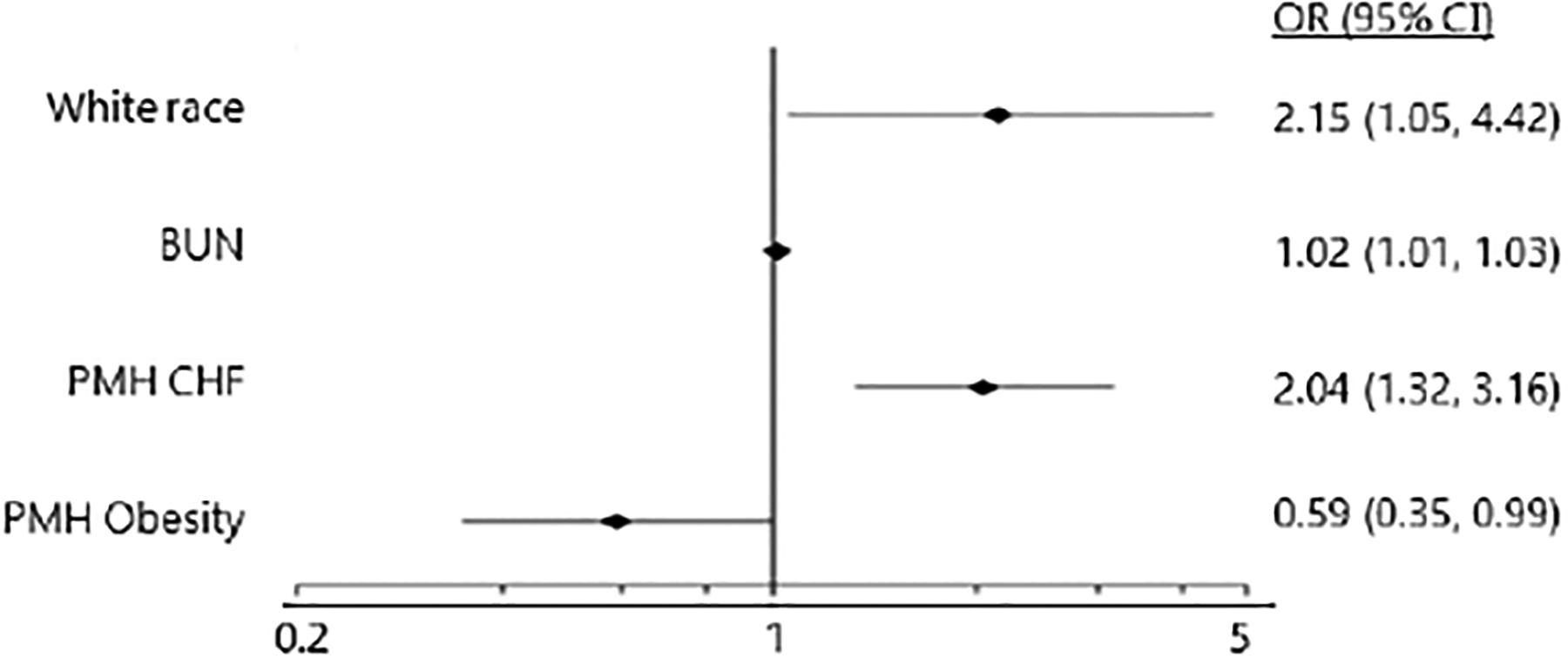
Multivariable Logistic Regression Model Comparing Late Readmissions to No Readmission in 30 Days (Reference Group). Reported odds ratios are adjusted for race, BUN upon admission, history of CHF, and history of obesity. Abbreviations: BUN = blood urea nitrogen; CHF = congestive heart failure; PMH = past medical history; OR = odds ratio; CI = confidence interval.

Of 1050 ACS patients who were not readmitted within 7 days, 128 (12.19%) were readmitted late (8–30 days after discharge) while 922 (87.81%) were not readmitted within the first 30 days (Table 1). Relative to non-readmissions, late readmissions were more likely to be in females, white patients, or patients requiring ICU admission during index hospitalization (Table 1). History of congestive heart failure (CHF) was a significant independent predictor of late readmission (C-statistic=0.65 and Hosmer-Lemeshow p-value = 0.69, Figure 4).

## Discussion

In this study, the purpose was to compare early and late readmissions in patients referred to BRIDGE following hospitalization with a principal discharge diagnosis of ACS (unstable angina, STEMI, NSTEMI) and identify clinical predictors of early and late readmission in this population. Three key findings were identified. First, there was a sex disparity with respect to readmission timing in this population. Second, principal discharge diagnosis of NSTEMI, past medical history of atrial fibrillation, and ICU admission were all significant independent predictors of early readmission among all studied patients. Finally, of all studied patients except early readmissions, history of CHF was predictive of late readmission.

Female ACS patients experienced greater rates of early readmission compared to males. This is consistent with other literature showing that women have a greater risk of 30-day readmission than men following hospitalization for acute myocardial infarction.^2,3,13–16^ However, one study reported that younger women (<65 years) had higher 30-day readmission rates following an acute myocardial infarction compared to men,^2^ but this same study, along with an additional study,^1^ showed that readmission timing within 30 days was similar between sexes. Of ACS patients readmitted within 30 days, we found that females had roughly twice the odds of early readmission relative to men, suggesting a sex disparity in readmission timing. While it has been noted that females have worse outcomes following hospitalization for acute myocardial infarction with respect to mortality, length of stay, and readmissions through 1 year post-discharge, further research is needed to understand the implications of our finding in order to better inform discharge planning.^2,13,17–19^ Such a targeted approach may provide an avenue for minimizing early readmissions that are potentially more preventable than late readmissions.^5,6^

Among all patients studied, we observed that having a past history of atrial fibrillation, principal discharge diagnosis of NSTEMI, or requiring ICU admission during index hospitalization were all significant independent predictors of early readmission. Atrial fibrillation has been shown to be associated with 30-day readmissions in several contexts including the following: development of atrial fibrillation during hospitalization for acute myocardial infarction, ACS patients with comorbid atrial fibrillation, new-onset postoperative atrial fibrillation, and past history of atrial fibrillation in patients hospitalized with STEMI.^20–24^ Similarly, other studies have shown that 30-day readmission rates are greater for NSTEMI compared to STEMI patients.^25,26^ While our results were concordant with these studies, our study is one of the first to limit the analysis to the first 7 days (rather than first 30 days). These three characteristics likely represent a combination of recurrent and difficult-to-control conditions (e.g., rate control in atrial fibrillation) and patients with more complex underlying disease or comorbidities (NSTEMI and ICU admission).^27–29^ Awareness of these early readmission predictors can potentially aid healthcare providers in effectively screening for patients who may require greater medical attention prior to discharge to prevent early readmission.

Finally, we observed that history of CHF was predictive of late, but not early, readmission. An abundance of literature has shown a direct relationship between history of CHF and 30-day readmission following an ACS.^15,30^ However, we were unable to find any studies demonstrating a differential risk of readmission in the first 7 days versus 8–30 days following discharge for an ACS in patients with history of CHF. Recognizing predictors of late readmission in ACS patients and how they differ from early readmission predictors can aid healthcare providers in addressing the varying needs of patients when creating post-discharge plans aimed at minimizing a patient’s risk of 30-day readmission.

Reduction of 30-day readmissions following acute myocardial infarction has been an area of interest for many health services researchers and health systems partly because of the Center for Medicare and Medicaid Services’ Hospital Readmission Reduction Program, which penalizes hospitals with greater risk-adjusted readmission rates greater than average readmission rates for particular conditions.^8^ While much research has been done to examine predictors of 30-day readmission in acute myocardial infarction patients, research investigating predictors of early and late readmission in the ACS population is limited. Such predictors of early and late readmission may provide health systems with additional information to guide readmission-reduction efforts on early readmissions that may be more preventable.^5,6^ For example, as ICU admission was a significant predictor of early readmission in this study population, health systems seeking to minimize early readmissions following ACS hospitalization may find it beneficial to consider increased referral to post-ICU clinics following patient discharge.^31,32^ Additional research is warranted to thoroughly characterize groups of acute coronary syndrome patients who may have greater susceptibility to readmission in a potentially more preventable time period (e.g., the first week of discharge). Moreover, future research could investigate predictors of early and late readmission in populations with other discharge diagnoses (cardiac and non-cardiac). Such work could help investigators determine if certain characteristics are associated with increased likelihood of early readmission, regardless of discharge diagnosis.

This study has important strengths. Since the BRIDGE registry has been maintained for more than 10 years, we had a large sample of ACS patients and a considerable number of these patients were readmitted within 30 days of discharge. Additionally, as data were abstracted manually by trained data abstractors, we did not need to rely on patient recall for any information. Finally, we had access to a comprehensive amount of health information for each patient given the way data was abstracted from the electronic medical record.

There are also limitations of this analysis. As this is an observational study based on retrospective data, causality cannot be determined. Furthermore, the results of this study should be applied cautiously to other populations, as the BRIDGE registry may not be reflective of populations with different proportions of younger/older individuals, males/females, and individuals of varying racial/ethnic background. Information bias via misclassification must also be considered, since patients with multiple active conditions had to be categorized into a single principal discharge diagnosis. Lastly, because this registry is maintained within one institution, the registry may underreport readmission to outside sites.

In summary, among ACS patients referred for short-term transitional care follow-up who were readmitted within 30 days, early readmissions were more likely in females, non-white patients, or patients requiring ICU admission during index hospitalization. Other early readmission predictors were past medical history of atrial fibrillation and principal discharge diagnosis of NSTEMI while history of CHF was predictive of only late readmission. The predictor differences between readmission groups likely represent differences in urgency at which patients require medical attention to prevent readmission. While the results of this study highlight the need for rapid outpatient follow-up when available, this study does not indicate if additional length of stay during index would decrease readmission. This needs to be explored in future studies. Understanding factors that influence readmission timing provides healthcare professionals with additional information to reduce readmissions, minimize financial penalties related to excessive readmissions, and improve quality of care for patients.

## Summary - Accelerating Translation

Acute coronary syndrome (ACS) is defined as any condition resulting from a sudden decrease in blood flow to the heart, such as heart attack. ACS is a common medical emergency requiring medical treatment, including hospital admission. Readmissions are also common among these patients, leading to increased health care costs. However, some readmissions may be avoidable, and some previous studies have suggested that readmissions occurring soon after hospital discharge (i.e. within one week) are more easily preventable. It has yet to be determined if any patient characteristics are associated with early readmissions (within 1 week of hospital discharge) compared to late readmissions (8–30 days after hospital discharge). The purpose of this study was to identify clinical predictors of early and late readmissions among ACS patients.

Patients hospitalized at Michigan Medicine due to ACS between April 2008 and November 2017 were included in this study. They were then divided into groups based on whether or not they were readmitted and the time to readmission (0–7 days after hospital discharge [early], or 8–30 days after hospital discharge [late]). Demographics, primary diagnosis, past medical history, readmission diagnosis, and hospitalization factors were compared between groups.

Of 1120 patients hospitalized following acute coronary syndrome, 198 (17.7%) patients were readmitted within the month following their discharge from the hospital. Most (128 patients; 64.6%) who were readmitted were readmitted late, between 8 and 30 days after hospital discharge. A smaller number (70 patients) were readmitted early, within the first 7 days of discharge (35.4%). Of all readmissions, early readmissions were more likely in females, non-white individuals, or patients requiring intensive care unit admission during hospitalization. Compared to patients not readmitted within 7 days, patients who were female, had history of a specific type of heart rhythm disturbance (atrial fibrillation), and a specific type of heart attack (non-ST segment elevation myocardial infarction) during the hospitalization, or required intensive care during their admission were more likely readmitted early. History of congestive heart failure was a predictor of late readmission when compared to patients not readmitted in 30 days.

The readmission predictors offered in this study may provide health systems with additional information to guide readmission-reduction efforts, potentially minimizing healthcare costs and improving quality of care for patients.

## Supplementary Material

Suppl_table_1

Suppl_table_2

Suppl_table_3

## Figures and Tables

**Table 1.** Baseline Characteristics of Early Readmissions versus No Readmission within 7 days and Late Readmissions versus No Readmission within 30 Days.

	Early Readmissions (0–7 days) (n=70, 6.25%)	No Readmissions within 7 days (n=1050,93.75%)	p-value	Late Readmissions (8–30 days) (n=128,12.19%)	No Readmissions within 30 days (n=922, 87.81%)	p-value
**Demographics**						
Age (years), mean ± SD	67.20 ± 15.13	63.54 ± 13.01	0.02	64.96 ± 12.87	63.34 ± 13.02	0.19
Female, n (%)	40 (57.1)	354 (33.7)	<0.001	50 (39.1)	304 (33.0)	0.17
Married, n (%)	31 (57.4)	514 (64.6)	0.29	62 (66.0)	452 (64.4)	0.77
Non-white, n (%)	12 (17.1)	133 (12.8)	0.57	10 (8.0)	123 (13.5)	0.09
Low SES, n (%)	14 (20.6)	226 (23.6)	0.32	31 (26.7)	195 (23.2)	0.40
**Principal Discharge Diagnosis, n (%)**						
Unstable angina	7 (10.6)	180 (18)	0.13	21 (16.9)	159 (18.1)	0.75
NSTEMI	42 (63.6)	535 (53.4)	0.11	64 (51.6)	471 (53.6)	0.67
STEMI	17 (25.8)	287 (28.6)	0.62	39 (31.5)	248 (28.2)	0.46
**Prevalence of comorbid conditions, n (%)**						
AF/Atrial Flutter	17 (24.3)	132 (12.6)	0.01	19 (14.8)	113 (12.3)	0.41
Aortic Stenosis	8 (11.4)	62 (5.9)	0.07	9 (7.0)	53 (5.7)	0.56
Cerebrovascular Disease	14 (20.0)	140 (13.3)	0.12	20 (15.6)	120 (13.0)	0.42
CHF	19 (27.1)	194 (18.5)	0.07	40 (31.3)	154 (16.7)	<0.001
Coronary Artery Disease	69 (98.6)	1021 (97.2)	0.50	125 (97.7)	896 (97.2)	0.76
Diabetes Mellitus	25 (35.7)	364 (34.7)	0.86	52 (40.6)	312 (33.8)	0.13
Hypertension	50 (71.4)	762 (72.6)	0.84	92 (71.9)	670 (72.7)	0.85
ICD/Pacemaker	8 (11.4)	67 (6.4)	0.10	7 (5.5)	60 (6.5)	0.65
Malignancy	12 (17.1)	163 (15.5)	0.72	25 (19.5)	138 (15)	0.18
Obesity (BMI ≥ 30)	15 (21.4)	240 (22.9)	0.78	21 (16.4)	219 (23.8)	0.06
Renal disease (acute or chronic)	20 (28.6)	223 (21.2)	0.15	42 (32.8)	181 (19.6)	0.001
Pulmonary disease	29 (41.4)	348 (33.1)	0.16	48 (37.5)	300 (32.5)	0.26
Vascular Disease	17 (24.3)	179 (17.0)	0.12	31 (24.2)	148 (16.1)	0.02
Charlson Comorbidity Index, median (25^th^, 75^th^)	5.60 (3.93, 7.13)	4.30 (3.00, 6.40)	0.002	5.10 (3.70, 6.90)	4.20 (3.00, 6.20)	0.001
**Patient characteristics during index hospitalization**						
Required ICU admission, n (%)	26 (37.7)	269 (25.7)	0.03	29 (23.0)	240 (26.1)	0.46
Total length of stay, mean ± SD	5.03 ± 3.59	4.45 ± 4.89	0.003	5.55 ± 4.91	4.30 ± 4.87	<0.001
Hemoglobin on arrival, mean ± SD)	12.54 ± 1.94	13.55 ± 3.10	<0.001	13.38 ± 6.91	13.58 ± 2.11	<0.001
Creatinine on arrival, mean ± SD	1.32 ± 1.22	1.21 ± 1.13	0.43	1.38 ± 1.27	1.18 ± 1.11	0.004
BUN on arrival, mean ± SD	25.68 ± 16.54	21.97 ± 12.74	0.01	26.33 ± 16.33	21.38 ± 12.06	<0.001
**Discharge Medications, n (%)**						
ACE inhibitor	40 (63.5)	632 (64.7)	0.85	72 (62.1)	560 (65.0)	0.53
ARB	11 (15.9)	139 (13.4)	0.56	23 (18.3)	116 (12.8)	0.09
P2Y_12_ Inhibitor	45 (64.3)	776 (74.0)	0.07	96 (75.0)	680 (73.9)	0.79
Aspirin	66 (94.3)	1004 (96.4)	0.38	121 (96.0)	883 (96.4)	0.84
Beta Blocker	61 (88.4)	893 (86.2)	0.61	111 (87.4)	782 (86.0)	0.67
CCB	11 (15.7)	176 (16.9)	0.80	19 (14.8)	157 (17.2)	0.51
DTI	1 (1.9)	3 (0.4)	0.23	0 (0.0)	3 (0.4)	>0.999
Nitrate	27 (50.0)	458 (57.5)	0.28	59 (62.1)	399 (56.8)	0.33
Statin	65 (94.2)	979 (95.0)	0.78	117 (93.6)	862 (95.1)	0.46
Warfarin	11 (15.7)	111 (10.7)	0.20	18 (14.2)	93 (10.2)	0.17
Xa inhibitor	0 (0.0)	16 (2.0)	0.62	3 (3.2)	13 (1.9)	0.40
**Readmission Diagnoses, n (%)**						
Recurrent ACS	7 (10.0)	-	-	14 (10.9)	-	-
CHF	7 (10.0)	-	-	14 (10.9)	-	-
Other cardiac diagnosis	25 (35.7)	-	-	57 (44.5)	-	-
Other non-cardiac diagnosis	28 (40.0)	-	-	37 (28.9)	-	-
All-cause mortality at 180 days post-discharge, n (%)	9 (12.9)	54 (5.3)	0.008	22 (17.9)	32 (3.5)	<0.001

ACE = angiotensin converting enzyme; ACS = acute coronary syndrome; ARB = angiotensin II receptor blocker; BMI = body mass index; BUN = blood urea nitrogen; CCB = calcium channel blocker; CHF = congestive heart failure; DTI = direct thrombin inhibitor; ICU = intensive care unit; NSTEMI = non-ST segment elevation myocardial infarction; SES = socioeconomic status; STEMI = ST-segment elevation myocardial infarction; 25^th^ = 25^th^ percentile; 75^th^ = 75^th^ percentile.
