# Supplementary figures and images for: Predictors of Early (0–7 Days) and Late (8–30 Days) Readmission in a Cohort of Acute Coronary Syndrome Patients
Source: Int J Med Stud. Author manuscript; Available in PMC 2022 Apr 19. (PMC9017796; doi:10.5195/ijms.2022.1058)

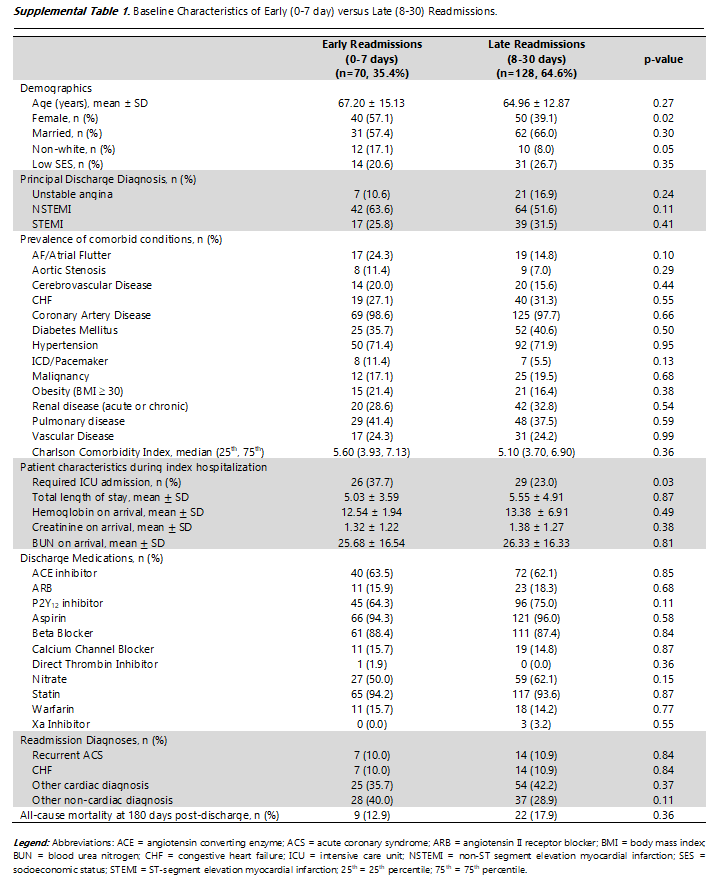

Supplement: Suppl_table_1 [file NIHMS1795617-supplement-Suppl_table_1.png]

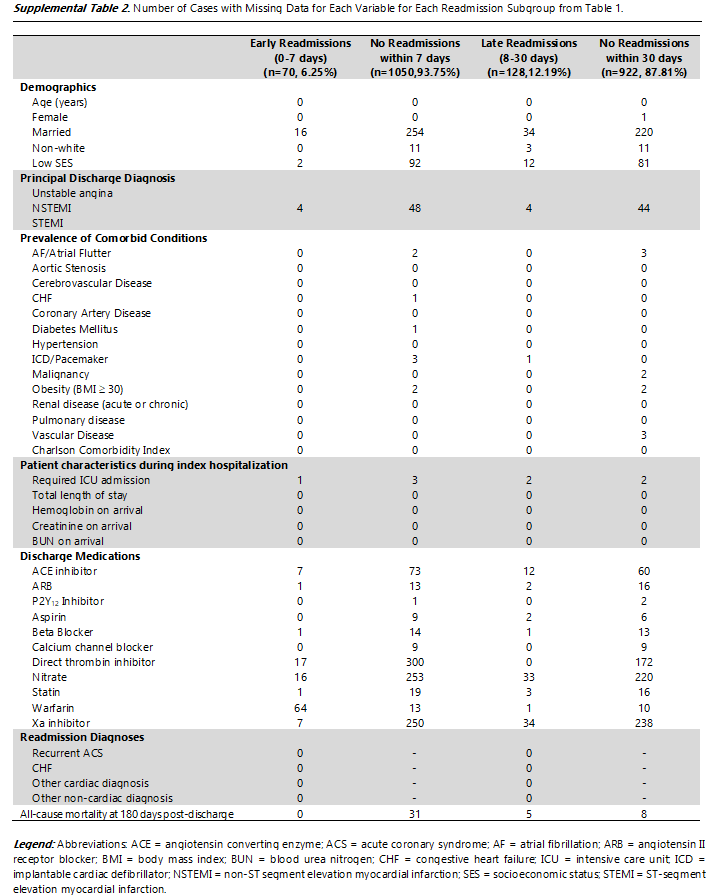

Supplement: Suppl_table_2 [file NIHMS1795617-supplement-Suppl_table_2.png]

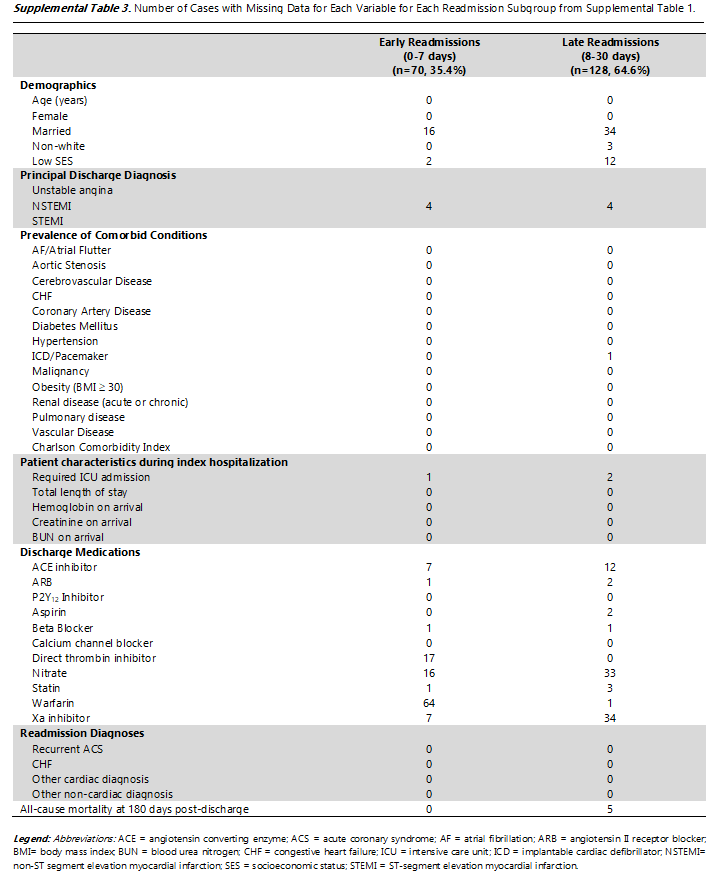

Supplement: Suppl_table_3 [file NIHMS1795617-supplement-Suppl_table_3.png]
